# Multifunctional biomimetic hydrogel dressing provides anti-infection treatment and improves immunotherapy by reprogramming the infection-related wound microenvironment

**DOI:** 10.1186/s12951-024-02337-3

**Published:** 2024-02-28

**Authors:** Xiaogang Bao, Shicheng Huo, Zhenhua Wang, Shengyan Yang, Linyun Dou, Yifei Liu, Jian Huang, Chang Cai, Bin Fang, Guohua Xu

**Affiliations:** 1 Department of Orthopedic Surgery, The Spine Surgical Center, Second Affiliated Hospital of Naval Medical University, Shanghai, 200003 China; 2 Department of Laboratory Medicine, Second Affiliated Hospital of Naval Medical University, Shanghai, 200003 China; 3 Department of Pharmacy, Second Affiliated Hospital of Naval Medical University, Shanghai, China; 4 Department of Orthopedics, the First Affiliated Hospital of Zhejiang Chinese Medical University, Hangzhou, 310000 China

**Keywords:** Infection-associated wounds, Bioactive glass nanoparticles, Magnesium, Curcumin, Immunomodulation

## Abstract

The advancement of biomaterials with antimicrobial and wound healing properties continues to present challenges. Macrophages are recognized for their significant role in the repair of infection-related wounds. However, the interaction between biomaterials and macrophages remains complex and requires further investigation. In this research, we propose a new sequential immunomodulation method to enhance and expedite wound healing by leveraging the immune properties of bacteria-related wounds, utilizing a novel mixed hydrogel dressing. The hydrogel matrix is derived from porcine acellular dermal matrix (PADM) and is loaded with a new type of bioactive glass nanoparticles (MBG) doped with magnesium (Mg-MBG) and loaded with Curcumin (Cur). This hybrid hydrogel demonstrates controlled release of Cur, effectively eradicating bacterial infection in the early stage of wound infection, and the subsequent release of Mg ions (Mg^2+^) synergistically inhibits the activation of inflammation-related pathways (such as MAPK pathway, NF-κB pathway, TNF-α pathway, etc.), suppressing the inflammatory response caused by infection. Therefore, this innovative hydrogel can safely and effectively expedite wound healing during infection. Our design strategy explores novel immunomodulatory biomaterials, offering a fresh approach to tackle current clinical challenges associated with wound infection treatment.

**Supplementary Information:**

The online version contains supplementary material available at 10.1186/s12951-024-02337-3.

## Introduction

The largest organ of the human body, the skin, plays a primary defensive role against external environmental threats [1]. Infections that permeate this barrier through wounds may cause severe inflammation, adversely affecting healing and potentially leading to life-threatening conditions [2]. Thus, it is crucial to develop innovative, multifunctional bioactive materials that can eradicate infections and enhance wound healing and tissue regeneration. Various types of dressing materials, such as nanofiber films and hydrogels, have been designed for this purpose [3, 4]. Hydrogels are known for their high permeability, biocompatibility and moisturizing qualities, positioning them as promising materials for treating infectious wounds [5, 6]. However, traditional hydrogel dressings often lack inherent antibacterial properties, limiting their use to conjunction with antibiotics [7, 8]. Regrettably, long-term use of antibiotics may yield bacterial resistance and provoke adverse hepatic or renal side effects, impeding the wound healing process [9, 10]. Therefore, there is an urgent need to develop new types of hydrogel dressings capable of taking effect without relying on antibiotics.

Infectious wounds render a host of complications owed to particular inflammatory conditions which obstruct the physiological sequence of wound healing and considerably challenge macrophages’ polarization from pro-inflammatory (M1) to anti-inflammatory (M2) phenotypes [11]. A surfeit of M1 macrophages accumulates within the wound, excreting pro-inflammatory cytokines like tumor necrosis factor-alpha (TNF-α) and producing reactive oxygen species [12]. This action consecutively induces oxidative stress and a cellular damage microenvironment within the wound, resulting persistent inflammation and vascular alterations. This sequence culminates in impeded wound healing, amplified inflammatory mediators and a perpetuated detrimental cycle [13–15]. Consequently, manipulating the immune microenvironment and fostering angiogenesis are crucial for healing infectious wounds. Immune modulation triggers the expression of transforming and proliferative cytokines, renowned for their angiogenesis promoting attributes [16]. However, accomplishing in-situ immune modulation-induced angiogenesis poses a challenge. Therefore, steering the wound microenvironment by governing macrophage heterogeneity to accomplish immune modulation transpires as a proactive strategy to remedy chronic wounds instigated by bacterial infection.

Recently, researchers have pointed out that nanodrugs based on natural products have potential therapeutic effects on various inflammation and infectious diseases [17–19]. Natural substances, such as curcumin (Cur), have been utilized to remedy a broad spectrum of illnesses, including cancer, bacterial infections, metabolic disorders, and autoimmune diseases [20–22]. Its potential effects are believed to be mediated via its properties of anti-inflammation, antioxidation, and immune regulation [23]. Curcumin, a natural polyphenolic compound, has a molecular weight of approximately 368.38 g/mol. Although it is a hydrophobic molecule with poor solubility in water, it significantly dissolves in polar solvents like ethanol, methanol, and dimethyl sulfoxide [24, 25]. Nonetheless, its clinical application has been inherently constrained by its low bioavailability [26, 27]. Mesoporous bioactive glass (MBG), a third-generation biomaterial, has attracted significant interest in the nanomaterials arena, credited to its unique attributes including extraordinary tissue regeneration and exceptional stability [28–30]. This class of materials modifies the extracellular matrix milieu by generating solubilization and degradation products that effectuate specific biological activities. Of particular note, soluble ions such as silica and calcium are thought to stimulate and excite cells at the implant site, thereby promoting tissue repair [31, 32]. Moreover, the liberation of active ions, such as magnesium (Mg) and strontium (Sr) ions, curbs the secretion of inflammatory cytokines by macrophages, thereby mitigating the inflammatory process [33, 34]. Among a large range of potential contenders, magnesium ions exert influence on macrophage polarization and back tissue repair. Furthermore, MBG is differentiated by its porous framework and substantial surface area which are indispensable for efficient local drug delivery.

Traditional synthetic hydrogel materials, such as chitosan, alginate, hyaluronic acid, polyethylene glycol, and mercaptosuccinic acid, exhibit toxicity, limited histocompatibility, labor-intensive production processes, and induce severe skin irritation [35, 36]. Moreover, the synthesis of hydrogels typically necessitates the utilization of toxic cross-linking agents like acrylic acid, polyvinyl alcohol, and glutaraldehyde, which possess cytotoxic properties and are challenging to neutralize or eliminate within the body [37]. Consequently, numerous researchers have endeavored to overcome these drawbacks by devising biopharmaceutical carriers [38]. Extracellular matrix materials (ECMs) have been documented to exhibit biocompatibility and possess the ability to replicate the natural cellular microenvironment [39]. They offer chemical and mechanical cues that guide cellular adhesion, proliferation, and differentiation, thereby facilitating the repair and regeneration of corresponding tissues [40]. Furthermore, our prior investigations have demonstrated the efficacy of skin extracellular matrix in promoting the healing process of skin [41].

**Scheme 1:**
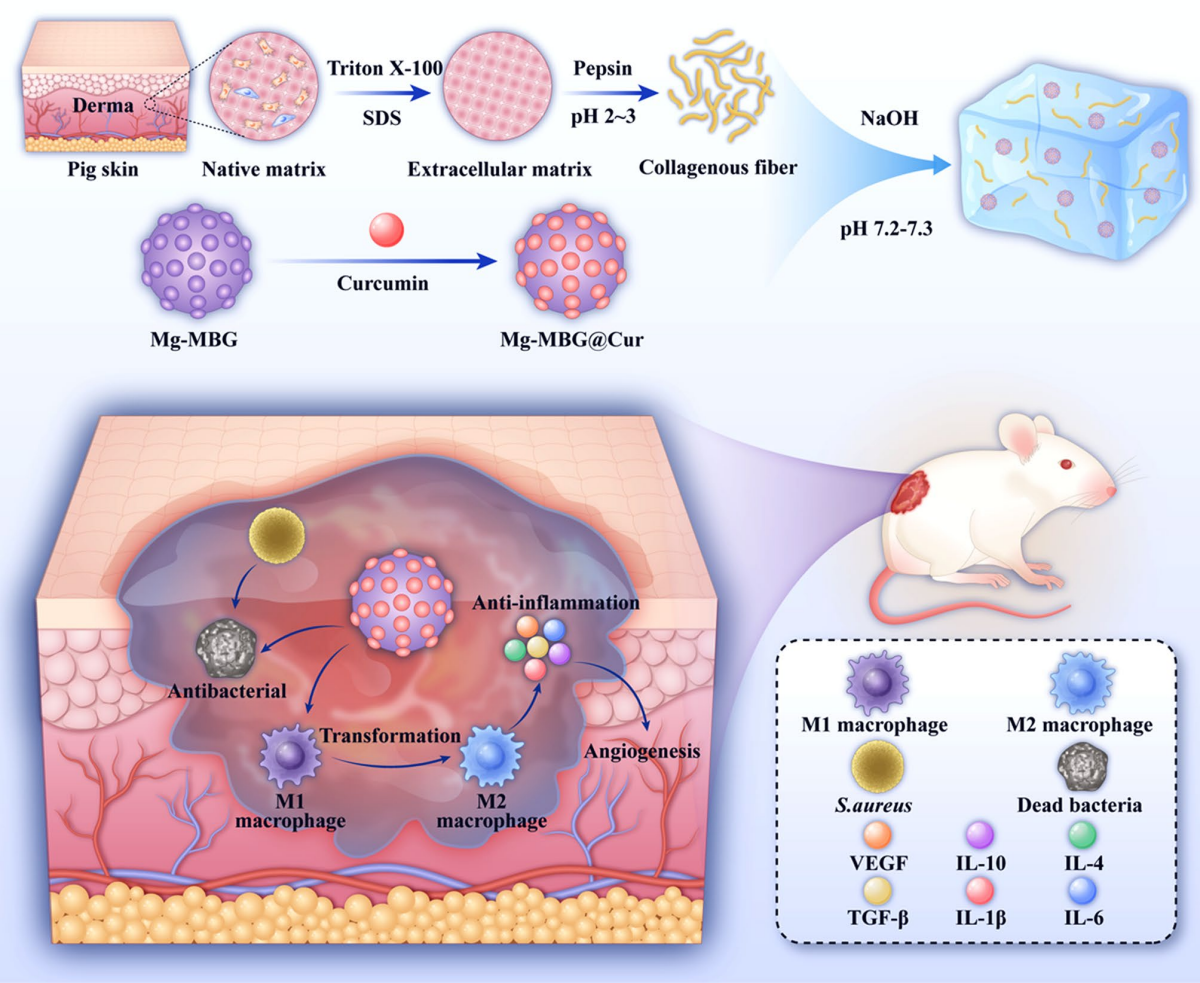
(**a**) Schematic of PADM@MgC hydrogel synthesis and (**b**) promotes the healing of infectious wounds through PADM@MgC’s antibacterial and anti-inflammatory properties

In this research, we devised and synthesized a unique hydrogel dressing encapsulating Cur-loaded Mg-MBG (Mg-MBG@Cur). During the initial stage, the incorporated Cur is released to counter infection and related inflammatory responses by impairing bacterial cell membranes. Following infection eradication, the Mg bioactive glass discharges Mg^2+^, further establishing an anti-inflammatory microenvironment that fosters vascularization and tissue restoration. In addition, these composite nanoparticles are evenly incorporated into a porcine-derived extracellular matrix-based hydrogel (PADM@MgC, Scheme 1). Results demonstrate that this PADM@MgC hydrogel materially reduces the nanoparticles’ toxicity and hastens wound healing. Mechanical and chemical signals on extracellular matrix (ECMs) mimic the cellular microenvironment, assisting in cellular adherence, proliferation, and differentiation, thus advancing homologous wound healing.

## Results and discussion

### Characterization of hydrogel

**Fig. 1:**
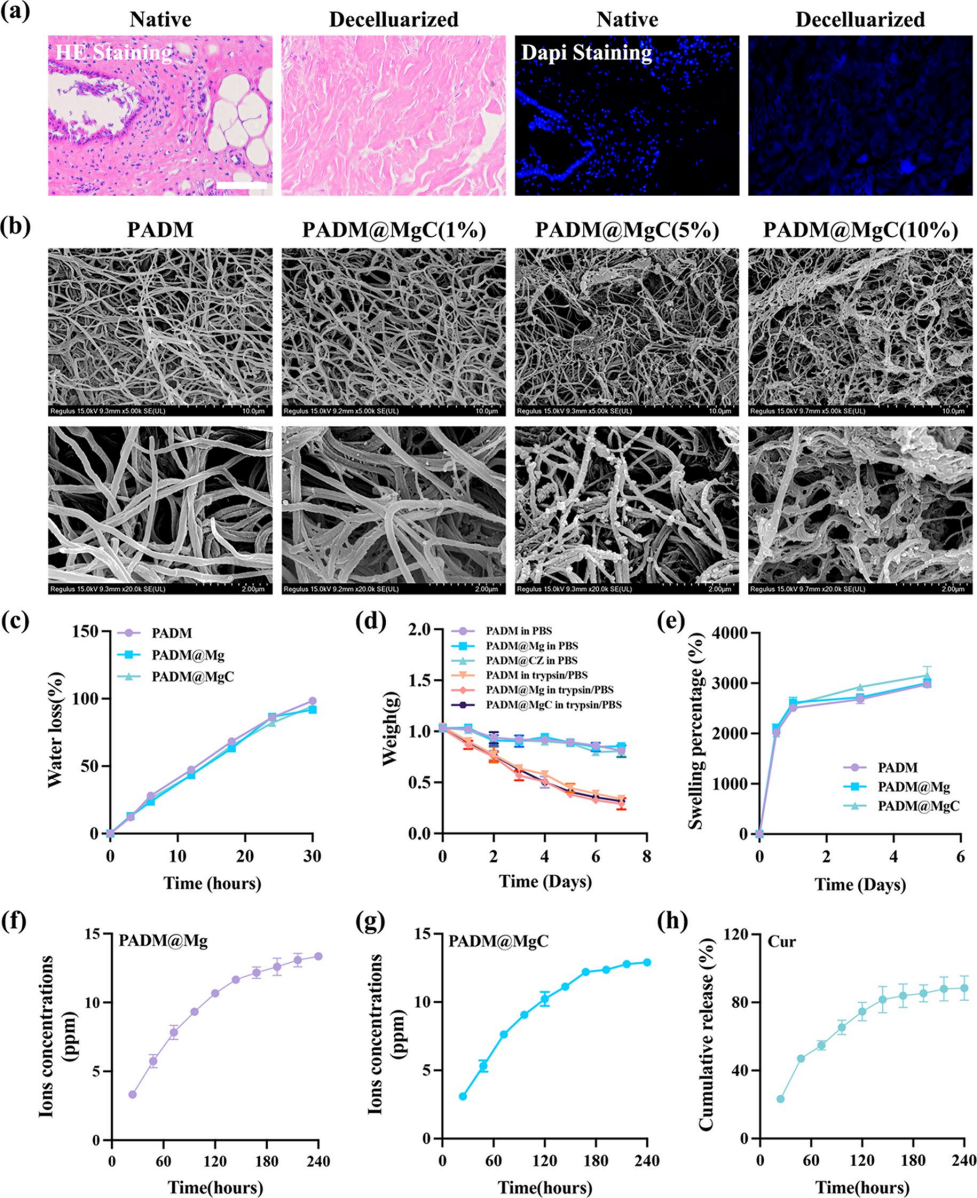
Preparation and Characterization of PADM@MgC hydrogels. (**a**) HE and DAPI stainings of the porcine skin extracellular matrix showing that cells are completely cleared. Scale bar: 500 μm. (**b**) SEM results for different PADM@MgC hydrogels (1%, 5%, and 10%). (**c**) PADM PADM@Mg, and PADM@MgC hydrogels exhibited negligibly different water retentions. (**d**) PADM, PADM@Mg, and PADM@MgC hydrogels degraded with or without trypsin. (**e**) Swelling percentage. (**f**, **g**) Concentration of released Mg^2+^ of the (**f**) PADM@Mg and (**g**) PADM@MgC hydrogels. (**h**) Cumulative release of Cur in PADM@MgC hydrogels

Hematoxylin and Eosin (HE) along with DAPI staining unequivocally demonstrated the complete removal of porcine skin cells while preserving the extracellular structures, as shown in Fig. 1a. The quantitative analysis of DNA revealed that the DNA content in the fresh skin group was 1044.22 ± 51.38ng/mg, while in the decellularised skin group it was 10.05 ± 3.53ng/mg, as depicted in Fig. S1. The DNA content of the decellularized group showed a significant decrease compared to the fresh group and demonstrated a statistically significant difference (*P < 0.0001*). Additionally, the DNA residues in the decellularized group were all below the internationally accepted standard of 50 ng/mg.

Fig. 1b displays representative Scanning Electron Microscopy (SEM) images of PADM and PADM@MgC (PADM containing MgC nanoparticles) at varying concentrations (1%, 5%, and 10%). Echoing the structural attributes of Extracellular Matrix (ECM) and tendon hydrogels, both PADM and PADM@MgC hydrogels exhibited a reticular, densely packed fibrous architecture. This suggests that pepsin-digested extracellular collagen effectively reassembles into collagen-like fibers. Additionally, SEM analyses substantiated the successful integration of Mg-MBG into the PADM@MgC hydrogels. However, it was observed that higher concentrations of Mg-MBG particles, particularly at 10%, led to a discernible alteration in the original structure of PADM, which might have implications for its functional properties. Furthermore, the fibrous structure inherent in PADM is conducive to wound healing. Therefore, alterations resulting from changes in particle concentration may adversely impact wound repair. Hence, for subsequent experiments, we opted for PADM@MgC comprising 5% of Mg-MBG particles as the designated experimental group.

The comparative analysis of water retention capabilities among various hydrogels is depicted in Fig. 1c. Notably, both hydrogel types demonstrated comparable water loss, fully dehydrating after around 30 h at a steady temperature of 37 °C. This similarity indicates that the integration of Mg-MBG into PADM hydrogels does not compromise their water retention efficiency. Consequently, PADM hydrogels are likely to effectively maintain hydration in wound environments, potentially enhancing the duration and quality of wound healing. Additionally, these hydrogels may possess an inherent capacity to absorb wound exudates, further contributing to an optimized wound healing process [42].

Trypsin-assessed degradation of PADM and AgNP-PADM hydrogels is presented in Fig. 1d, indicating varied rates over time. Degradation rates of PADM, PADM@Mg (PADM containing Mg-MBG nanoparticles) and PADM@MgC hydrogels in PBS were discovered to be 14.13 ± 1.65%, 14.44 ± 0.76% and 13.67 ± 1.41% at the 7-day mark. Notably, degradation occurred at an amplified rate within trypsin, leading to significant differences (PADM group: 76.87 ± 2.8%; PADM@Mg group: 79.55 ± 1.4%; PADM@MgC group: 77.86 ± 2.4%). The degradation behaviour showcases consistency, potentially due to the primary adsorptive nature of the nanoparticles and PADM hydrogels, with negligible influence by enzyme degradation [43]. In contrast, when trypsin solution was utilized, a notable escalation in the rate of hydrogel degradation was observed compared to the baseline PBS solution. A plausible explanation for this observation could be centered around the initial conversion of decellularized porcine skin particles into assorted collagen fibers in the course of gel preparation, coupled with the presence of specific trypsin-degradable protein constituents within PADM hydrogels [44]. Our findings propose that assorted proteases released during tissue injuries may expedite the degradation of other hydrogel protein components, in turn promoting the discharge of nanoparticles.

Subsequently, an assessment was conducted to determine the swelling capacities of various hydrogels, revealing that the interaction between PADM and PBS led to a swift augmentation in mass, surpassing 2500% of the initial mass (Fig. 1e), and this increase was sustained for a duration of 5 days. Furthermore, the inclusion of bioglass did not compromise PADM’s commendable water absorption capability and satisfactory water retention, thus exhibiting potential in absorbing wound exudate and consequently expediting the process of wound healing.

Upon submerging PADM@Mg and PADM@MgC in phosphate buffer across varying durations, the resulting alterations in Mg ion concentration were meticulously documented (as shown in Fig. 1f and g). Notably, the Mg ion concentration in both PADM@Mg and PADM@MgC exhibited a gradual increase from 6 h, extending up to 7 days, characterized by a sustained release pattern. Fig. 1h presents the drug release profile of PADM@MgC when immersed in PBS for specific periods, revealing a relatively rapid release rate within the initial 48 h, followed by a consistent slow-release trajectory. Remarkably, over a period of 240 h (10 days), Cur attained a total release of approximately 94%.

### Antibacterial efficacy and mechanism of PADM@MgC hydrogel

**Fig. 2:**
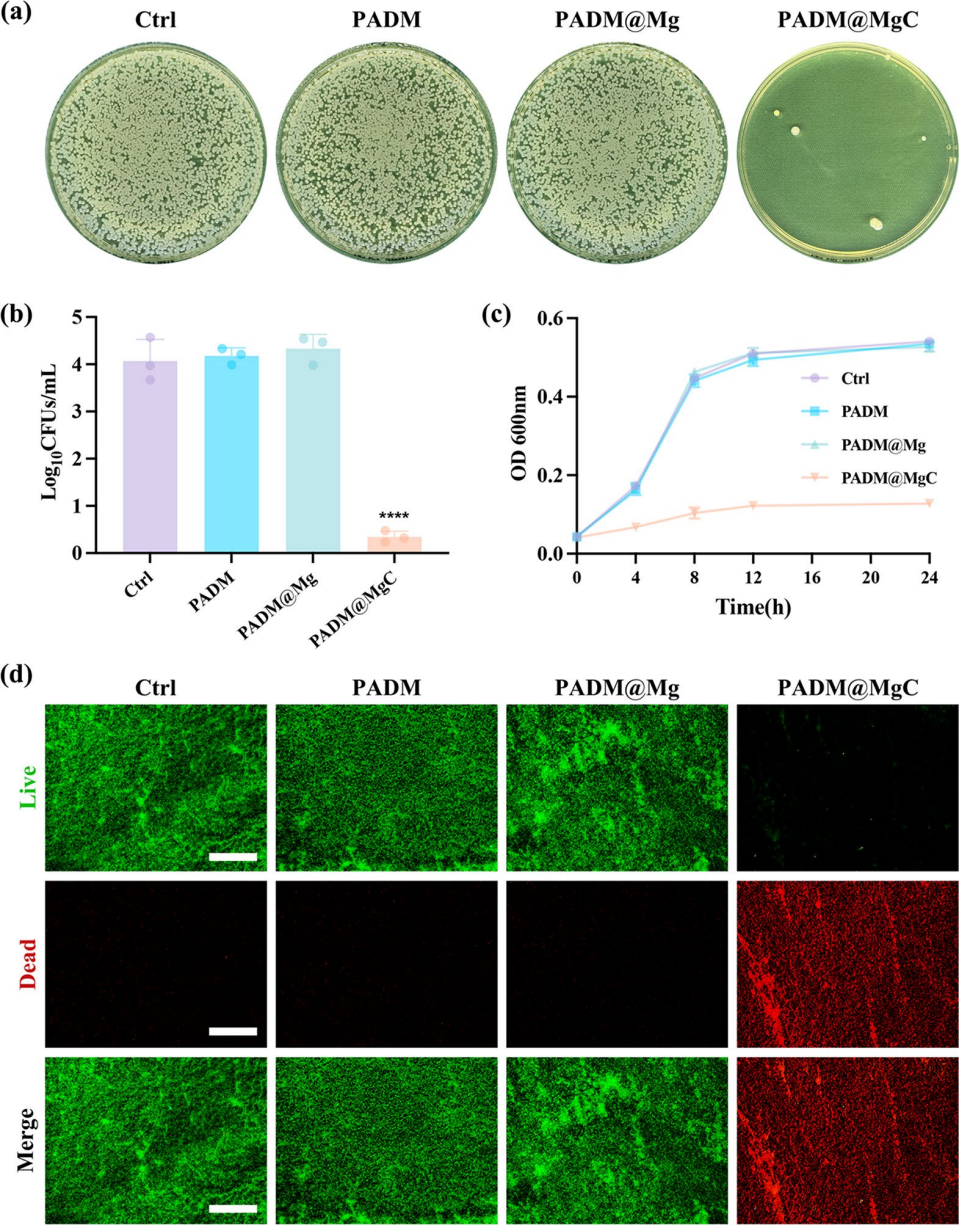
antibacterial effect of various hydrogels. (**a**) Representative images of the bacterial colonies formed by the bacteria cultured with various hydrogels after 24 h. (**b**) Quantitative analyses of the bacterial populations after treatment with various hydrogels. (**c**) The time-kill kinetics results of various hydrogels against bacteria. (**d**) The survival of bacteria after various treatments was assessed using live/dead staining. Live bacteria are indicated by green fluorescence, while dead bacteria are represented by red fluorescence. Scale bar: 100 μm. Results are presented as mean ± SD (*N* = 3). ****P < 0.0001

To evaluate the antibacterial potential of PADM@MgC hydrogel, MRSA was employed as the test organism. Initially, bacterial colonies were enumerated using the standard plating method (SPM) (Fig. 2a). In the PADM@MgC group, a dramatic decline in bacterial survival rate was observed, plummeting from 100.15% ± 6.45% to a mere 3.85% ± 6.39%. In contrast, the survival rates in other groups remained relatively unchanged, as illustrated in Fig. 2b. Subsequently, the bacterial growth curves under various conditions were charted, based on the initial absorbance readings of the bacterial suspensions (Fig. 2c). The data revealed that while PADM@Mg hydrogel exerted a modest inhibitory effect on bacterial proliferation, the growth rate of bacteria in the presence of PADM@MgC hydrogel was markedly diminished. Further analysis involved staining cells from different treatment groups with a live/dead dye, followed by observation under a fluorescence microscope, as depicted in Fig. 2d. A pronounced increase in bacterial mortality (indicated in red) was evident in the PADM@MgC group post-treatment. Conversely, the control group and the PADM@Mg group exhibited a substantial presence of viable bacteria (shown in green), underscoring the relatively weaker bactericidal effect of the PADM@Mg hydrogel. Collectively, these results demonstrate the potent bactericidal activity of PADM@MgC hydrogel, highlighting its effectiveness in bacterial eradication.

**Fig. 3:**
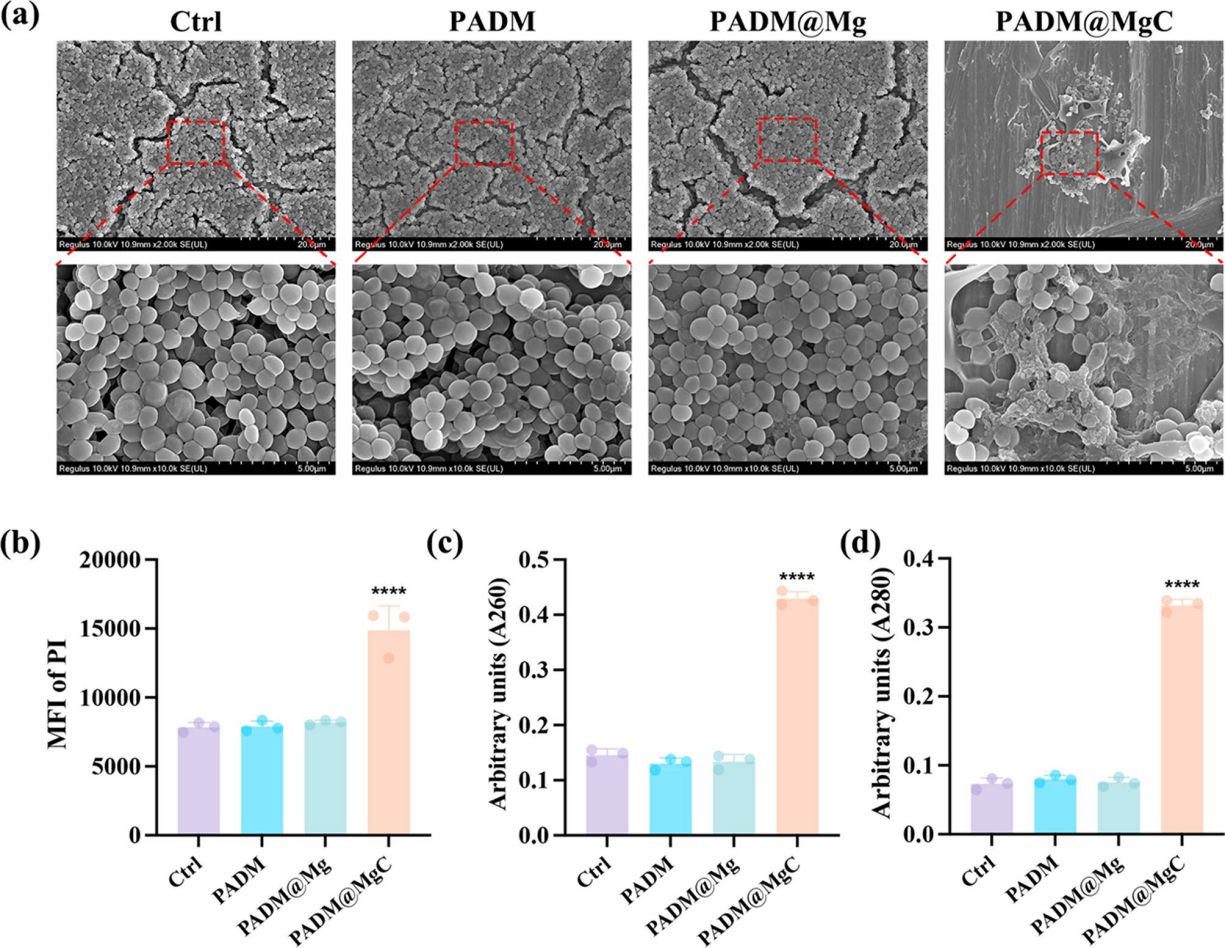
Research on the mechanism of in vitro antimicrobial action. (**a**) SEM images of bacteria colonies and morphology after treatment with various hydrogels. (**b**) Various hydrogels-induced membrane damage assessed from PI uptake. Leakage of cellular contents following exposure to different PADM-based hydrogels was examined by measuring the absorbance of extracellular material at (**c**) 260 nm (A260) and (**d**) 280 nm (A280), which correspond to nucleic acids and proteins, respectively. Results are presented as mean ± SD (*N* = 3). ****P < 0.0001

To delve into the antibacterial action of PADM@MgC, we initially employed SEM to observe the morphological alterations in MRSA. The SEM images revealed that untreated MRSA maintained normal morphology, with clear boundaries and intact membranes (Fig. 3a). In stark contrast, MRSA exposed to PADM@MgC exhibited severe membrane damage and alteration. In addition, a comparative analysis between the control group and the PADM@MgC-treated group highlighted drastic differences. While the control group’s bacteria retained intact membranes, those treated with PADM@MgC displayed pronounced membrane damage, coupled with apparent leakage of intracellular contents. This observation underscores PADM@MgC’s capacity to directly compromise the integrity of bacterial cell membranes.

Subsequently, we employed PI to investigate the potential of PADM@MgC hydrogel to disrupt bacterial cell membranes. The findings revealed a considerable increase in the permeability of bacterial membranes upon treatment with PADM@MgC hydrogel, as depicted in Fig. 3b. Notably, the PI fluorescence intensity in the PADM@MgC group showed significant elevations of 46.67%, 45.52%, and 43.64% compared to the control, PADM, and PADM@Mg groups, respectively. This data strongly suggests that the PADM@MgC hydrogel markedly disrupts bacterial cell membranes. In parallel, MRSA treated with different hydrogels underwent absorbance analysis at 260 nm (A260) and 280 nm (A280) for nucleic acids and proteins, respectively. The PADM@MgC group exhibited a pronounced increase in absorbance at these wavelengths compared to the control, PADM, and PADM@Mg groups, as illustrated in Fig. 3c and d.

These findings collectively demonstrate the potent antibacterial effects of PADM@MgC hydrogel in vitro. Specifically, during bacterial infection, the acidic microenvironment leads to the degradation of PADM@MgC hydrogel. This process results in the sustained release of Curcumin, which acts as an efficacious bactericidal agent. This activity disrupts the bacterial cell membranes, induces leakage of cellular contents, alters biochemical processes, and ultimately precipitates bacterial demise.

### Macrophage behavior

Effective wound repair hinges on a well-orchestrated inflammatory response, ensuring the restoration of damaged tissue. In this complex process, the immune system plays an indispensable role [45, 46]. However, when the immune response becomes dysregulated or excessively amplified, it can exacerbate tissue damage rather than facilitate healing. Thus, achieving a balanced and appropriate inflammatory response is critical for successful wound repair and treatment. Macrophages, pivotal players in the immune response, demonstrate remarkable versatility by polarizing into M1 and M2 phenotypes in response to varied stimuli [47]. This polarization is crucial; properly activated M1 and M2 macrophages can significantly enhance tissue repair processes. Conversely, inappropriate activation and polarization may lead to heightened inflammatory responses, impeding effective wound healing. The host immune response undeniably plays a significant role in hydrogel-mediated wound repair. Given the importance of this interaction, we turned our focus to investigating the immunomodulatory effects of PADM@MgC. Understanding how this hydrogel influences macrophage behavior and the broader immune response is key to harnessing its potential in promoting optimal wound healing.

#### Immunofluorescence staining and flow cytometry

**Fig. 4:**
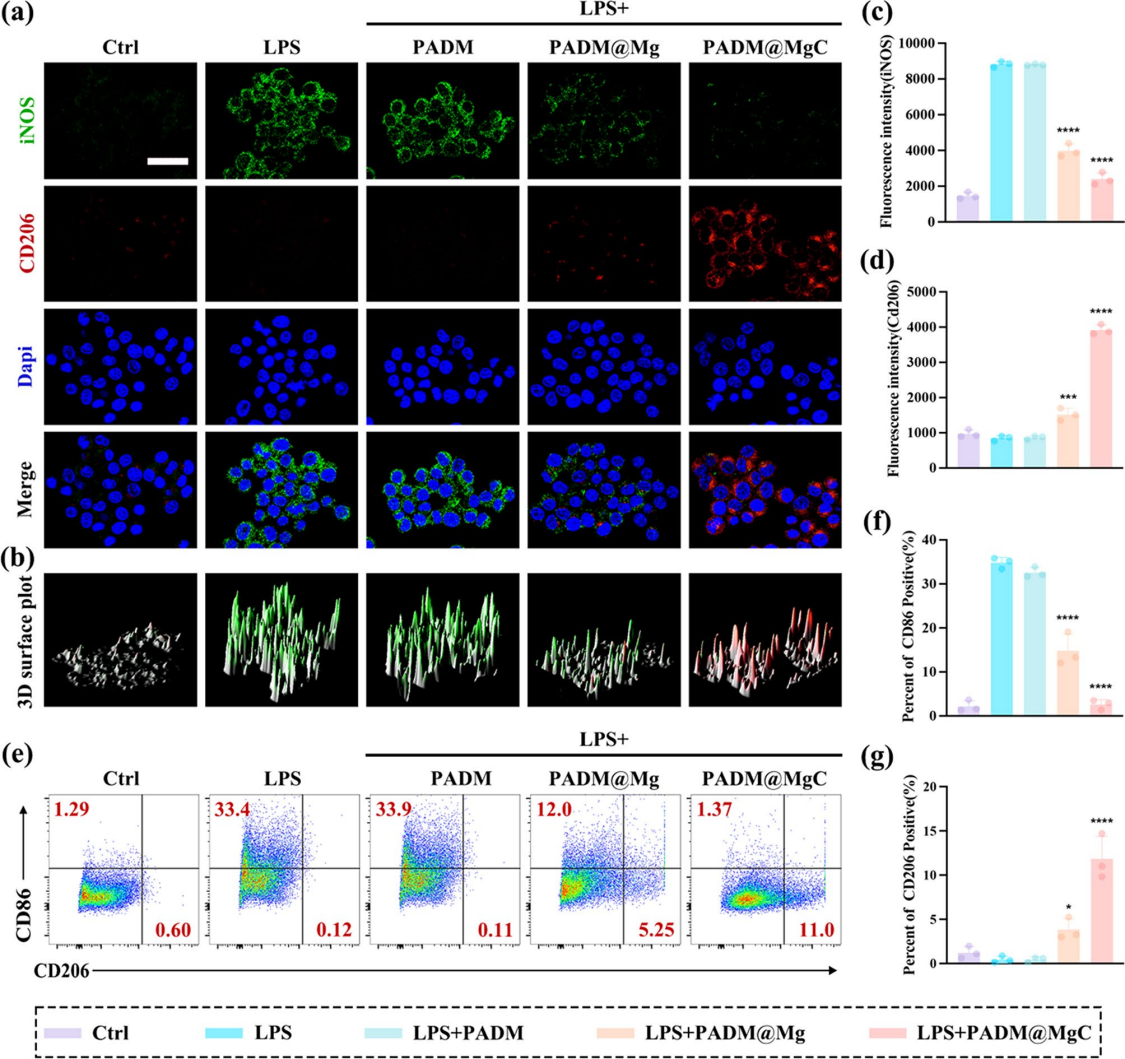
Macrophage behavior on various PADM-based hydrogels. (**a**) After different hydrogel treatments, macrophages were detected for the expression of M1 (iNOS: green) and M2 (CD206: red) marker proteins using CLSM. Scale bar: 20 μm. (**b**) 3D surface plot of iNOS (green) and CD206 (red). (**c**) Statistical analysis of iNOS fluorescence intensity. (**d**) Statistical analysis of CD206 fluorescence intensity. (**e**) The surface markers CD86 and CD206 of RAW 264.7 after different hydrogel treatments were analysed by flow cytometry. (**f**) Positive CD86 and CD206 (**g**) cell percentage. Results are presented as mean ± SD (*N* = 3). ***P < 0.001; ****P < 0.0001

RAW264.7 macrophages were co-cultured with different hydrogels in the presence of LPS for one day, and the immunofluorescence effect was assessed. The polarization of macrophages by PADM@MgC was evaluated by analyzing the expression of M1 (iNOS) and M2 (CD206) markers. In Fig. 4a, the results showed that cells highly expressed iNOS (green fluorescence) after LPS treatment, while PADM@Mg and PADM@MgC significantly reduced the fluorescence intensity of iNOS (Fig. 4c), meaning they could inhibit the expression of iNOS. On the other hand, the fluorescence intensity of CD206 showed the opposite trend (Fig. 4d). Compared to the LPS group, PADM@Mg and PADM@MgC significantly increased the fluorescence intensity of CD206 (red fluorescence). From the co-localization 3D simulated peak map, it can also be observed that after LPS treatment, the peak of iNOS (green) was dense and high, whereas the peak of CD206 was dominant after PADM@MgC co-treatment (Fig. 4b). It is evident that the PADM@Mg and PADM@MgC hydrogel treatments had a noticeable impact on the expression of iNOS and CD206. The shift in fluorescence intensity indicates their potential to modulate the immune response.

The flow cytometry analysis revealed findings that were in harmony with the immunofluorescence results. Specifically, in both PADM@Mg and PADM@MgC groups, a notably low proportion of CD86-negative cells (M1 marker) and a high proportion of CD206-positive cells (M2 marker) were observed. In a comparative analysis (Fig. 4e-g), the percentage of cells expressing CD86 was significantly lower in the PADM@Mg (12.0%) and PADM@MgC (1.37%) groups than in the LPS group (33.4%) and PADM group (33.9%). Conversely, the proportion of CD206-positive cells was markedly higher in the PADM@Mg (5.25%) and PADM@MgC (11.0%) groups compared to the LPS group (0.12%). These results indicate a substantial correlation between macrophage polarization towards an M2 anti-inflammatory phenotype and the treatment with PADM@Mg and PADM@MgC hydrogels. The differential expression of CD86 and CD206 in these groups underscores the potential of PADM@Mg and PADM@MgC hydrogels in directing macrophage activity towards a more reparative, anti-inflammatory response, integral for effective healing processes.

#### RNA-Seq analysis

**Fig. 5:**
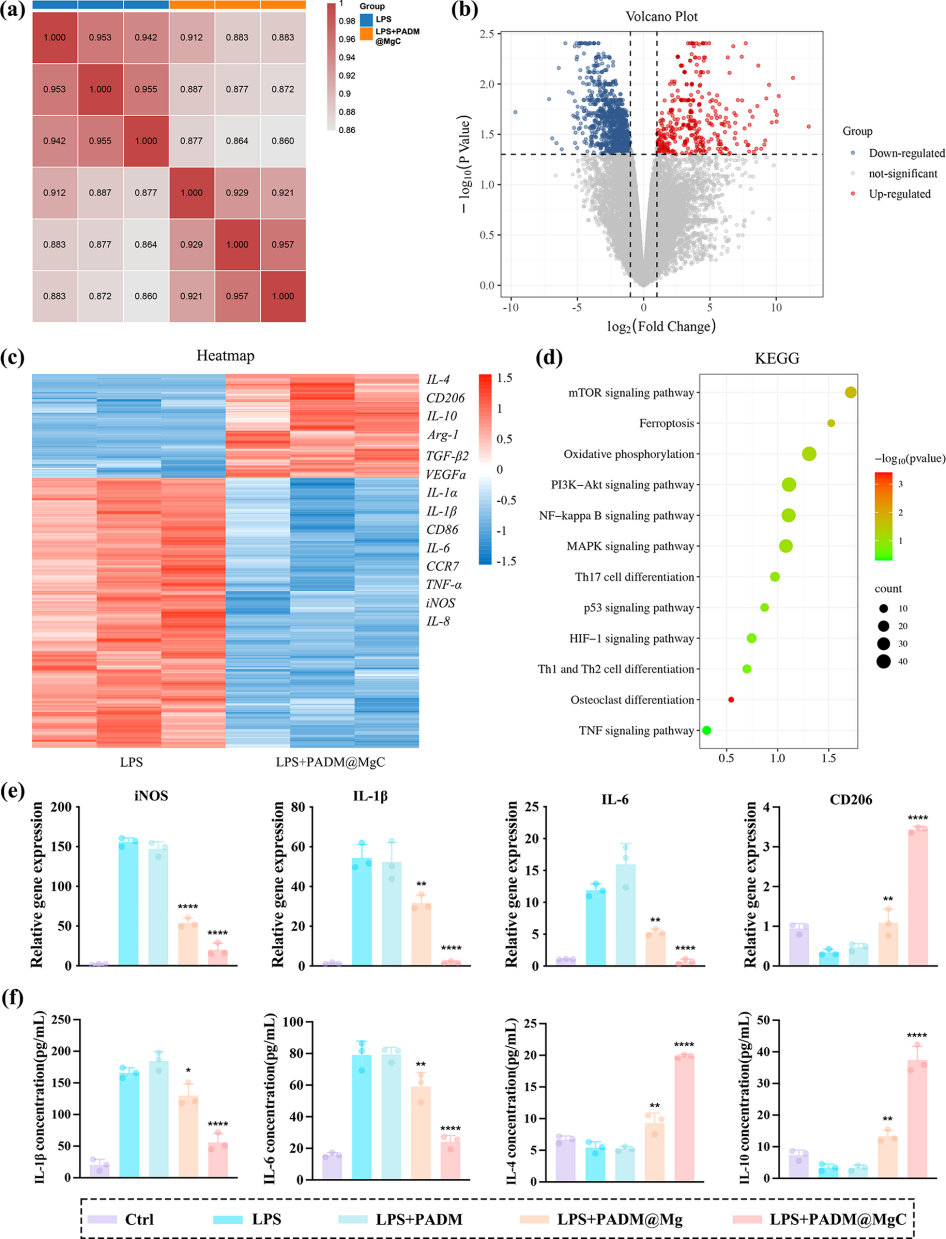
Gene expression analysis of macrophage cultured with various PADM-based hydrogels. (**a**) Correlation analysis of gene expression levels between LPS and LPS + PADM@MgC treated macrophages. (**b**) Volcano plot displaying the differentially expressed genes (fold change ≥ 2 and *p* < 0.05) in macrophages co-cultured with LPS and PADM@MgC. (**c**) Microarray heatmap illustrating the fold changes in expression levels of specific genes. (**d**) The top 12 downregulated pathways were analyzed using the KEGG pathway method. (**e**) Real-time PCR to detect macrophage polarization and expression of inflammation-related genes. (**f**) ELISA to detect inflammatory factors in cell supernatants. Results are presented as mean ± SD (*N* = 3). *P < 0.05; **P < 0.01; ****P < 0.0001

Macrophages treated either with LPS alone or in combination with PADM@MgC were subjected to comprehensive mRNA expression analysis via RNA-Seq. This approach was crucial in exploring the immunomodulatory mechanisms potentially induced by PADM@MgC hydrogel. Correlation analyses indicated strong agreement between samples from each treatment group, suggesting comparable gene expression across the groups (Fig. 5a). Subsequently, we identified differentially expressed genes (DEGs) based on the following criteria: *p* < 0.05 and fold change (FC) ≥ 2. A total of 1386 DEGs were discovered in the PADM@MgC group on day 5 compared to the control group, comprising 501 up-regulated and 885 down-regulated genes (Fig. 5b). The resulting heatmap vividly illustrates variations in gene expression, particularly in surface markers indicative of distinct macrophage phenotypes, between the LPS and LPS + PADM@MgC groups (Fig. 5c). Notably, PADM@MgC treatment was associated with upregulation of M2 macrophage markers, including CD206 and IL-10. In contrast, markers characteristic of the M1 phenotype, such as IL-1β, IL-6, and tumor necrosis factor-α (TNF-α), were significantly downregulated. This pattern points to a distinct shift in macrophage polarization towards the M2 phenotype. KEGG pathway enrichment analysis further illuminated the specific signaling pathways implicated in the macrophage phenotype transition (Fig. 5d). The analysis revealed a significant inhibition of M1-related inflammatory pathways, including mTOR, NF-κB, MAPK, and TNF-α signaling, following PADM@MgC treatment.

#### RT-PCR and elisa

To corroborate the RNA-Seq results, qPCR was employed. Following a 24-hour co-cultivation period, we observed that the expression of canonical M1 macrophage markers, including IL-1β, IL-6, IL-8, iNOS, and CCR7, was notably reduced in the PADM@MgC group compared to the group treated solely with LPS. In contrast, the expression of the M2 macrophage marker CD206 was found to be upregulated, aligning with the RNA-Seq data (Fig. 5e). In line with the RT-PCR findings, the secretion levels of pro-inflammatory cytokines such as IL-1β and IL-6 were also substantially diminished in the PADM@MgC group (Fig. 5f). This reduction further validates the anti-inflammatory potential of PADM@MgC, underscoring its role in modulating macrophage polarization towards a less inflammatory, M2 phenotype. These findings underscore the capability of PADM@MgC hydrogel to modulate macrophage phenotypes, especially by fostering an anti-inflammatory M2 state while concurrently suppressing the pro-inflammatory M1 state. These findings emphasize the ability of PADM@MgC hydrogel to modulate macrophage phenotypes, especially by promoting anti-inflammatory M2 state while inhibiting pro-inflammatory M1 state. The significant downregulation of key inflammatory markers and pathways suggests that PADM@MgC hydrogel may represent a promising therapeutic approach, exerting favorable effects on wound healing.

The phenotypic features of macroscopic research are determined by their inducers, properties, and surface markers. In this study, we loaded Cur into Mg-MBGs and then encapsulated them with PADM. We first explored the regulatory effect of PADM@MgC on macrophages and found that it has a profound impact on the M2 phenotype transition of macrophages. PADM@MgC treatment led to a decrease in LPS-induced M1 subtype macrophages and an increase in M2 subtype macrophages. Based on these findings, we concluded that PADM@MgC can reverse the macrophage phenotype changes induced by LPS. By comparing with the ICP results, we found that the Mg ion concentration in PADM@MgC is 6.38 mg/L. Previous studies have demonstrated that free Mg ions can suppress the expression of inflammatory genes. Furthermore, numerous studies have also indicated that Cur has a strong anti-inflammatory effect, which has been further confirmed in our research.

### In vitro migration and angiogenesis studies

**Fig. 6:**
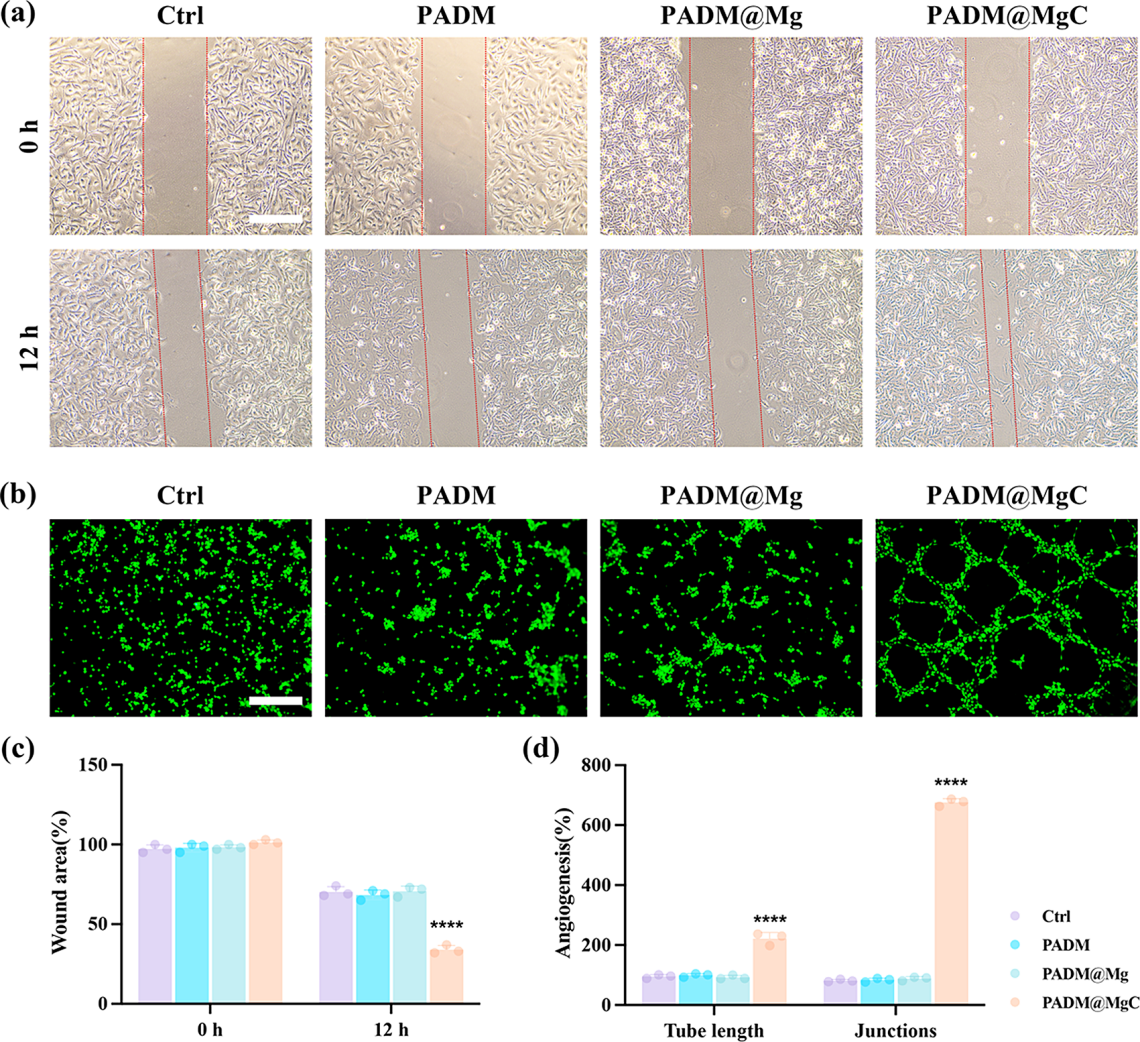
In vitro cell migration and angiogenesis behavior of various PADM-based hydrogels. (**a**) Different migration abilities of HUVECs after coculturing with the extract of various PADM-based hydrogels. Scale bar: 100 μm. (**b**) Quantification of gap closure ratio of different groups. Scale bar: 100 μm. (**c**) Fluorescence images of tube formation experiment via coculturing HUVECs with the extract of various PADM-based hydrogels. (**d**) Quantifications of junctions and tube length in each group. Results are presented as mean ± SD (*N* = 3). ****P < 0.0001

We evaluated the impact of PADM, PADM@Mg, and PADM@MgC infusions on cell migration using a scratch assay. The findings indicated a varying degree of enhancement in cell migration across the PADM, PADM@Mg, and PADM@MgC treated groups, relative to the untreated control group. Notably, the Cur-PADM@MgC group exhibited the most pronounced effect, achieving a significant 1.73-fold decrease in the residual scratch area after 12 h, compared to the control (Fig. 6a and c). This suggests the superior efficacy of Cur-PADM@MgC in facilitating cell migration.

The enhancement of angiogenesis is crucial for the restoration of tissue integrity. We then assessed the angiogenic influence imparted by PADM@MgC employing a tube formation assay with HUVECs. The cells were cultured with DMEM (serving as the control), or with extracts of PADM, PADM@Mg, or PADM@MgC for a duration of 12 h. Relative to the baseline control, a significant upsurge in tubular networking, as evidenced by the counts of junctions and elongation of tubes, was observed across all treatments, with a pronounced effect in the PADM@MgC cohort (Fig. 6b). Statistical analysis illustrated that the PADM@MgC extract augmented the average tube length to 3.23 times that of the control, outstripping the PADM and PADM@Mg increments by factors of 3.18 and 3.03, respectively (Fig. 6d). In a similar vein, the mean nodule formation experienced a surge of 7.72, 7.46, and 7.28 times relative to the control, PADM, and PADM@Mg aggregates, accordingly (Fig. 6d).

Studies have indicated that administering curcumin by gavage can enhance neovascularization and wound healing in mice with diabetic foot ulcers. Additionally, the local application of 1% turmeric extract gel has been found to have a beneficial impact on angiogenesis [48]. Furthermore, there is no substantial variance in the effects of various administration routes of curcumin on wound healing. The results of this investigation indicated that the rates of cell migration and angiogenesis were significantly enhanced in cells treated with PADM@MgC, which is advantageous for the healing of wounds, although the exact mechanism requires further exploration.

### In vitro biocompatibility

**Fig. 7:**
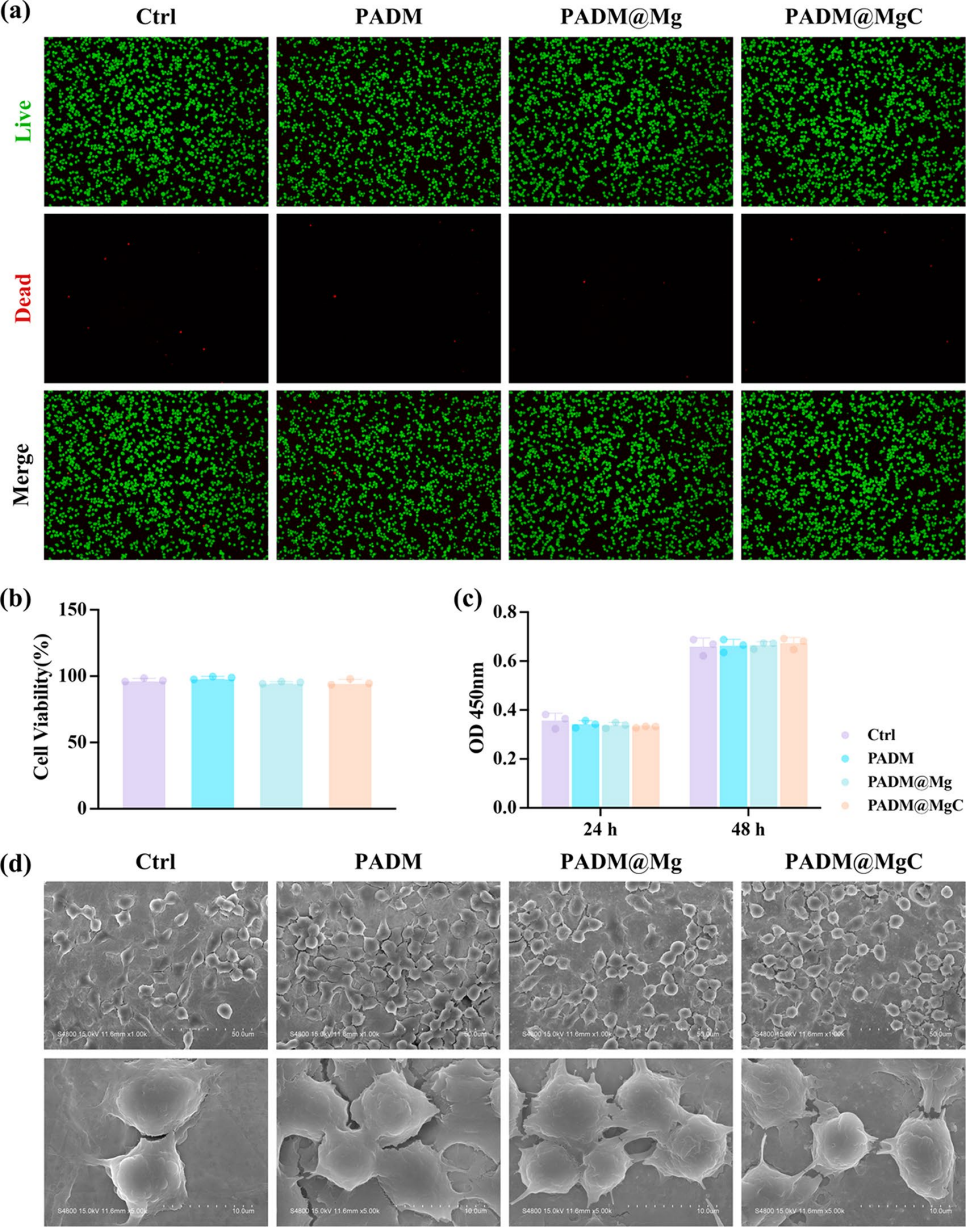
The cytocompatibility of different PADM-based hydrogels in vitro. (**a**) Confocal microscopy images of a live/dead assay of cells co-cultured with several PADM-based hydrogels for 24 h. Scale bar: 200 μm. (**b**) Quantitative analysis of rusults from (**a**). (**c**) Cell proliferation of PADM-based hydrogels. (**d**) SEM results of RAW 264.7 co-cultured with PADM-based hydrogels for 24 h

The biocompatibility of hydrogels is a vital parameter for their adoption in clinical settings as wound dressings. To this end, cell viability assays were conducted after culturing cells on various hydrogel compositions for a 24-hour period followed by live/dead staining procedures. Live cells exhibited green fluorescence, while the nuclei of nonviable cells emitted red fluorescence. Observations indicated a predominance of viable cells post-incubation on hydrogels-control, PADM, PADM@Mg, and PADM@MgC (Fig. 7a-b). Subsequent quantification via the CCK-8 assay reaffirmed the absence of significant cytotoxicity attributed to the hydrogels (Fig. 7c). SEM imaging further corroborated these findings by displaying cells in a state of healthy morphology upon hydrogel substrates (Fig. 7d).

### In vivo investigation on PADM@MgC for Infected Wound Healing

**Fig. 8:**
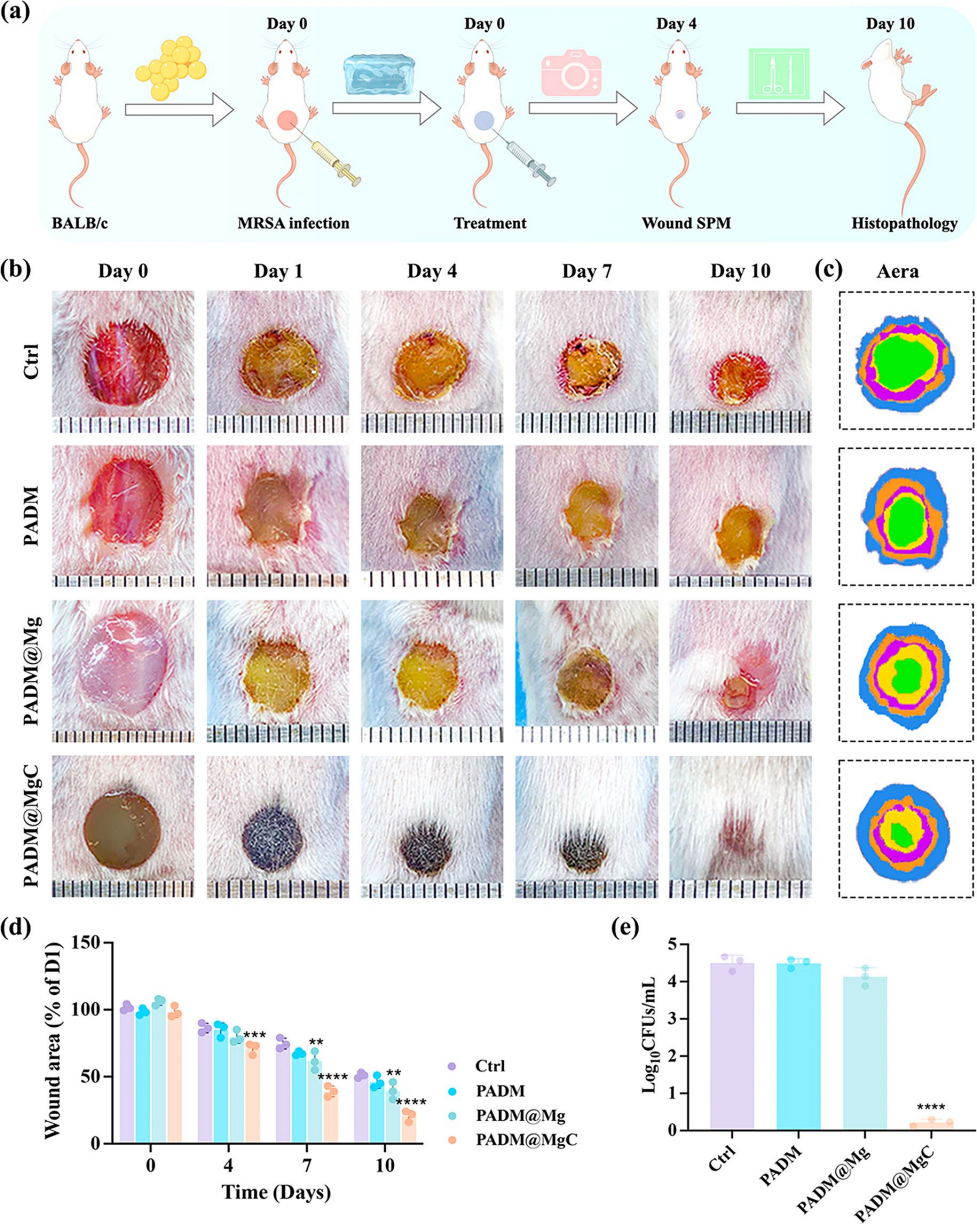
PADM@MgC demonstrates increased effectiveness in facilitating the healing of infected wounds in vivo. (**a**) Illustration of the model for mouse excisional wound infection and the experimental methods. (**b**) Representative photographic images of wounds in different groups after 0, 1, 4, 7, or 10 days of treatment. (**c**) Day 0 (blue), day 1 (orange), day 4 (purple), day 7 (yellow), and day 10 (green) explanatory photos of the wounds, as well as superimposed photos of treatment with various PADM-based hydrogels. (**d**) Area of unhealed wounds in each group at different time periods. (**e**) Bacterial counts in the wounds of the different treatment groups. Results are presented as mean ± SD (*N* = 3). **P < 0.01; ***P < 0.001; ****P < 0.0001

Prolonged non-healing of wounds may increase the risk of bacterial infection. Furthermore, this type of infection may compromise the immune function, thereby adversely affecting wound healing and ultimately leading to systemic infection. Therefore, expediting wound healing is imperative for preventing and treating infections. Next, we will utilize a murine excisional skin infection model to determine the impact of PADM@MgC hydrogel on wound healing (Fig. 8a).

With the progression of time, the infected wounds in each treatment group gradually underwent healing (Fig. 8b-c). However, significant variations were observed in the infection severity and rate of healing. Four days ago, the wounds of the control group, PADM group, and PADM@Mg group all exhibited evident infection, tissue necrosis, and discharge of pus. In contrast, the wounds of the PADM@MgC group remained consistently clean and dry throughout the entire observation period, showing no signs of obvious infection or suppuration. Furthermore, the mice in the PADM@MgC group demonstrated quicker wound healing (Fig. 8d), with only 17.1 ± 2.5% of the wounds remaining unhealed on the 10th day, followed by the PADM@Mg group (33.2% ± 1.6%), PADM group (42.5% ± 2.6%), and the control group (44.1% ± 2.1%). Additionally, on the 4th day, spreading plate method (SPM) was employed to evaluate the bacterial survival rate on the wounds after different treatments, showing a significant decrease in the bacterial survival rate following treatment with PADM@MgC hydrogel (Fig. 8e).

**Fig. 9:**
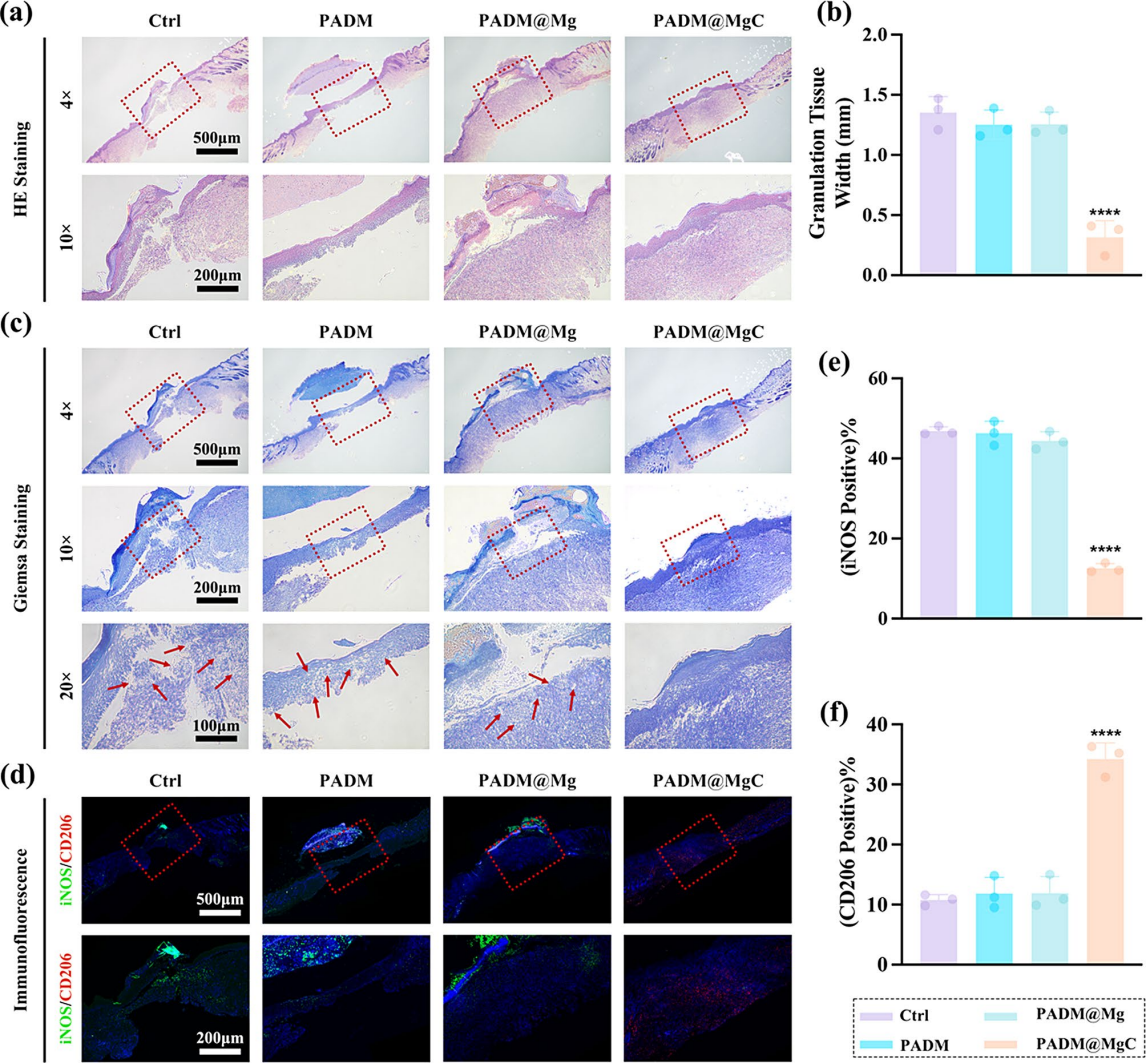
PADM@MgC hydrogel-induced acceleration of the wound repair. (**a**) Images of HE and Masson’s trichromatic staining of the infectious wounds of different groups on day 10. (**b**) Corresponding quantitative analysis of the granulation tissue width on Day 10. (**c**) Images of Giemsa staining of the infectious wounds of different groups on day 10. (**d**) Immunofluorescence staining for iNOS (M1-labelled) and CD206 (M2-labelled) was performed on tissue sections at the wound site on days 4 post-infection e, (**f**) Statistical analyses regarding the proportion of (**e**) iNOS- and (**f**) CD206-positive macrophages. Results are presented as mean ± SD (*N* = 3). ****P < 0.0001

Histological evaluations were undertaken through HE staining (Fig. 9a), with findings aligning with the statistical patterns observed in wound surface diminution. Moreover, the extent of granulation tissue formation among the mouse cohorts was quantified (Fig. 9b). Notably, the PADM@MgC-treated group exhibited accelerated wound closure, a trend that was most evident during the initial healing phases. Quantitative assessment of the granulation tissue thickness indicated a superior contraction rate in the PADM@MgC group relative to its counterparts. Effective wound repair is contingent upon vascularization to supply essential nutrients and oxygen. Consequently, angiogenic sufficiency is a critical component of the healing process. To gauge angiogenic activity, we assessed the expression levels of endothelial cell marker, cell adhesion molecule 1 (CD31), within the wound beds (Fig. S2). Comparative analysis indicated a heightened presence of CD31 in the wound tissues treated with PADM@MgC hydrogel. Collectively, these data point towards the notable wound-healing capabilities of PADM@MgC hydrogel. Further immunofluorescence analysis revealed that the PADM@MgC group had the lowest proportion of M1 macrophages (iNOS) (Fig. 9d and e) and the highest proportion of M2 macrophages (CD206) (Fig. 9d and f), confirming the in vitro results. These findings suggest that PADM@MgC triggers macrophage polarization towards an M2 phenotype, inducing an anti-inflammatory microenvironment, thereby promoting wound healing.

## Conclusion

Here, we developed a PADM@MgC hydrogel to enhance post-infectious wound healing by sequentially releasing Cur and Mg^2+^. The therapeutic effectiveness of PADM@MgC was optimized through (i) improved stability and bioavailability of Cur; (ii) anti-inflammatory, antimicrobial, and cell proliferation-promoting effects of Cur; (iii) Mg^2+^ improving cell migration and angiogenesis; (iv) Mg^2+^ and curcumin synergistically promote repolarization of M2 macrophages; (v) the hybridized hydrogel forming a physical barrier to prevent secondary damage; and (vi) the hybridized hydrogel achieving exudate absorption and antimicrobial PADM@MgC. Various functions of PADM@MgC have been demonstrated in vivo and in vitro. However, our study did not comprehensively examine the mobilization and function of other immune cells (e.g. DC cells, T cells, NK cells, etc.) in addition to macrophages during the initial stages of infected wound healing. Additionally, the molecular mechanisms of these processes have not been explored. In the next phase of our research, we will focus on these aspects in order to better understand the factors that promote infected wound healing through the use of biomaterials. Nevertheless, this novel diachronic bidirectional immunomodulatory hydrogel has good antimicrobial and wound healing effects and can be used to design and synthesize biomaterials that modulate immune responses according to the immune profile of the target disease.

## Materials and methods

### Synthesis of PADM@MgC hydrogel

The preparation protocol for Mg-MBG and Mg-MBG@Cur is comprehensively outlined in the Supporting Information. The porcine skin tissue, after a 3-hour rinse in sterile water, underwent multiple freeze/thaw cycles using liquid nitrogen. This was followed by agitation at 120 rpm and 25 °C to facilitate the removal of subcutaneous layers. The tissue samples were then treated with 0.1% Triton X-100 for 12 h and subsequently with 0.1% sodium dodecyl sulfate for 6 h. The final steps involved lyophilization, grinding into a fine powder, and digestion with pepsin in an acidic milieu (pH 2–3) for 10 min. Mg@C nanoparticles were introduced and rapidly mixed into the solution. The resulting gel was refrigerated at 4 °C and enzymatically digested over a 2-hour period until it achieved a translucent, viscous consistency. Subsequent steps included the addition of PBS to regulate osmotic pressure, followed by pH normalization to 7–8 using chilled 10 M NaOH. PADM@MgC hydrogels were then synthesized by incubating these gels at 37 °C for 20 min, followed by freeze-drying and compression. The final PADM@MgC hydrogels, with concentrations of 1%, 5%, and 10%, were determined based on the mass differential between the added nanoparticles and the freeze-dried hydrogel powder. In parallel, PADM@Mg hydrogels were synthesized using an identical process, except Mg-MBG were used in place of Mg@C nanoparticles for PADM@Mg preparation.

### Characterization of PADM@MgC hydrogels

Initially, Hematoxylin and Eosin (HE) and DAPI staining were conducted to evaluate the removal of cellular and nuclear debris. The DNA content of each set of skin tissue samples was analyzed using a DNA extraction kit, following the instructions in the manual. The DNA content of each set was measured using a UV spectrophotometer. Following this, the samples underwent dehydration through a critical point drying process. The structural characteristics of the hydrogels were then examined via scanning electron microscopy. Subsequently, the chemical composition of these materials was investigated using X-ray Photoelectron Spectroscopy (XPS), X-ray Diffraction (XRD), and Fourier-Transform Infrared (FTIR) spectroscopy.

### Water retention

The hydrogels PADM, PADM@Mg, and PADM@MgC were incubated at 37 °C, and their masses were periodically recorded until a stable mass was noted. The water retention capacity of these hydrogels was determined using the formula:

1 $${\rm{Water}}\,{\rm{retention}}\,{\rm{rate }} = {\rm{ W}}2/{\rm{W}}1{\rm{ }} \times {\rm{ }}100\%$$

Here, W2 denotes the hydrogel’s weight at each measured time point, while W1 represents its initial weight.

### Biodegradability

The biodegradability of PADM, PADM@Mg, and PADM@MgC hydrogels was determined under different conditions. The hydrogels were solidified in PBS and then incubated at 37 °C with or without trypsin. The solution was removed from the centrifuge tube, and the hydrogels were washed with PBS daily. Finally, the samples were dried and weighed.

### Hygroscopic properties

To investigate the hygroscopic properties of the hydrogel, 1 mL of the synthesized hydrogel was divided into thirds and subjected to lyophilization to obtain the initial dry weight (W0). Subsequently, the hydrogel was immersed in a 10 mL PBS solution and incubated at 37 °C for different time intervals (10 min, 30 min, 30 min, 30 min, and 30 min). At each time point, the hydrogel was removed, and its weight (Wt) was measured. The swelling ratio was then calculated using the formula:

2 $${\rm{Swelling}}\,{\rm{ratio }} = {\rm{ }}\left( {{\rm{Wt }} - {\rm{ W}}0} \right){\rm{ }}/{\rm{ W}}0{\rm{ }} \times {\rm{ }}100\%$$

### Ion release assessment

The investigation of ion release from PADM@MgC hydrogels involved placing an equal mass of hydrogel in 10 mL of simulated body fluid (SBF, PH1820, China). The medium was entirely replaced daily with fresh SBF to maintain consistent conditions. Copper and zinc ion concentrations were quantified using an inductively coupled plasma atomic emission spectrometer (X Series 2, Thermo Fisher Scientific, USA). For comparative analysis, control hydrogels PADM and PADM@Mg were subjected to identical testing procedures.

### Cells and bacteria

This study utilized mouse-derived macrophages (RAW264.7) and human umbilical vein vascular endothelial cells (HUVEC), both acquired from the Shanghai Institute of Cell Biology. These cells were cultured in DMEM medium, enriched with 10% fetal bovine serum (FBS) and 1% penicillin/streptomycin. Additionally, Methicillin-resistant Staphylococcus aureus (MRSA, ATCC 43,300) was procured from the American Type Culture Collection and propagated in tryptic soy broth (TSB; Hopebio).

### Preparation of sample extracts

The PADM, PADM@Mg, and PADM@MgC hydrogels were sectioned into 10 × 10 × 5 mm dimensions and submerged in DMEM supplemented with 10% FBS and 1% penicillin/streptomycin. The cultures were incubated at 37 °C for 24 h, after which the extracts were obtained and preserved at 4 °C for subsequent analysis.

### In vitro antibacterial tests

Frozen MRSA cultures were revived and incubated overnight in TSB at 37 °C on a shaker. This was followed by a dilution step at a 1:10,000 ratio, leading to further incubation until the cultures reached the logarithmic growth phase. Subsequently, bacterial suspensions with a concentration of 1 × 10^6^ CFU/mL, in a volume of 500 µL, were introduced to a variety of hydrogels for incubation. The influence of these hydrogels on bacterial growth was evaluated through growth curve assays. These experiments were conducted in triplicate and replicated thrice to ensure the robustness of the data. Following this, the bacteria were co-cultured with the different hydrogels for 24 h. Post-incubation, SPM and colony-forming unit (CFU) quantification were carried out on agar plates to further assess the interaction between the bacteria and the hydrogels. Additionally, we analyzed the antibacterial activity by live/dead staining. Briefly, we added 500 µL of combined dye (Syto9 and PI) in different samples and cultured them for 15 min. Bacteria were collected on glass slides and visualized under a fluorescence microscope.

### Membrane damage and leakage assessment

To detect membrane damage and leakage, bacteria and hydrogels were co-cultured for 24 h. Post-co-culture, SEM was employed to examine the morphological characteristics of the bacteria. In addition, the samples were stained with Propidium Iodide (PI) at a concentration of 2.5 µg/mL for 30 min. The fluorescence intensity of these stained samples was then measured at an excitation/emission wavelength of 535 nm/615 nm using a microplate reader. Subsequently, cell content leakage under various treatment conditions was evaluated. This involved centrifuging the bacterial supernatant post-co-culture at 8000 × g for 10 min, followed by measuring the absorbance at 260 nm for nucleic acids (A260) and 280 nm for proteins (A280) using a spectrophotometer. All experiments were conducted in triplicate to ensure consistency and accuracy.

### In vitro immunomodulatory evaluation

### Immunofluorescence

Briefly, RAW264.7 cells were incubated with PADM, PADM@Mg, or PADM@MgC hydrogels for one day. Subsequently, the cells were collected, fixed, and blocked before staining with antibodies against iNOS (ab210823) and CD206 (ab64693). Next, cells were incubated with corresponding secondary antibodies and counterstained with DAPI for 1 h. The cells were observed and photographed using CLSM.

### Flow cytometry assay

Flow cytometry, employing antibodies against CD86 and CD206, was utilized to assess the ratios of M1 and M2 macrophages. Macrophages were initially cultured with various hydrogels for a 24-hour period. This was followed by a 40-minute blocking phase and staining with allophycocyanin (APC)-conjugated CD206 and phycoerythrin (PE)/Cy7-conjugated CD86. The final steps involved washing the cells, resuspending them in 400 µL of PBS, and their subsequent evaluation using flow cytometry.

### Gene expression

To investigate gene expression profiles in macrophages, we undertook transcriptome sequencing analyses. RAW 264.7 cells, initially inoculated at 5 × 10^5^ cells per well, were subjected to a 24-hour incubation with diverse hydrogels in an LPS-enriched environment. Following this, total cellular RNA was meticulously extracted using TRIzol reagent, in strict accordance with the manufacturer’s instructions. The subsequent RNA-sequencing and bioinformatics analyses were proficiently conducted by Novel Biotech, Shanghai. This process facilitated the examination of differentially expressed genes (DEGs) for their involvement in KEGG pathways and gene ontology enrichment.

### RT-PCR and ELISA

RAW 264.7 cells were cultivated following the above outlined method and harvested after a 24-hour period. Total RNA was then meticulously extracted utilizing TRIzol. For accuracy in gene expression analysis, normalization was conducted against the housekeeping gene ACTB. Details of the primer sequences used are comprehensively listed in the Supplementary Information (Table S1). Furthermore, culture supernatants were harvested from macrophages post-incubation with various hydrogels. The concentrations of cytokines IL-1β, TNF-α, IL-6, and IL-10 in these supernatants were quantitatively assessed employing Enzyme-Linked Immunosorbent Assay (ELISA).

### Cell viability analysis

Cellular cytotoxicity was evaluated using a live/dead staining kit, applied as per the manufacturer’s guidelines. Additionally, to ascertain the biocompatibility of the hydrogels, a Cell Counting Kit-8 (CCK-8) assay was conducted. In this process, cells were co-cultured with different hydrogels at a density of 2 × 10^4^ cells per well. After 24 h, for live cell staining, cells were rinsed and incubated with calcein and PI for dead cells, for 30 min. Fluorescence microscopy was utilized to capture images of the stained cells. Concurrently, at designated intervals (24 and 48 h), the culture supernatant was removed, and 500 µL of prepared CCK-8 solution was added. Following an additional 2-hour incubation, absorbance readings at 450 nm were taken using a BioTek microplate reader. This procedure was replicated thrice for each experimental group to ensure consistency of results. Moreover, post-incubation with hydrogels, cells were harvested, centrifuged at 1500 rpm for 5 min, and subsequently fixed in 2.5% glutaraldehyde at 4°C overnight. The samples then underwent a meticulous dehydration process through a graded ethanol series (30%, 50%, 70%, 80%, 90%, and 100%), followed by freeze-drying. After being sputter-coated with platinum to enhance conductivity, the morphological characteristics of the cells were meticulously examined using scanning electron microscopy.

### In vitro migration study

Initially, HUVEC cells were seeded into 6-well plates and allowed to incubate for 24 h. Following this period, a precise line was drawn through each well’s center, after which the wells were gently washed twice with PBS. The cells were then exposed to extracts of PADM@MgC, PADM@Mg, and PADM, as well as complete medium for comparative purposes. To monitor cellular migration, images were captured at the outset and again after 12 h of treatment.

### Tube formation assay

Initially, each well of a 96-well plate was prepped with 50 µL of growth factor-reduced Matrigel, followed by a 30-minute incubation to ensure proper solidification of the Matrigel. Subsequently, 2 × 10^4^ HUVECs were carefully inoculated into each well containing the solidified Matrigel substrate. These wells were then divided and co-cultured with high-glucose DMEM infused with extracts from PADM@MgC, PADM@Mg, and PADM, alongside a complete medium serving as the control. Twelve hours post-incubation, the cells were stained using a calcein staining kit. Representative images capturing the tube formation were taken 6 h after treatment to document the cellular responses.

### Mouse wound model and treatment procedures

The Animal Ethics and Welfare Committee of Chang Zheng Hospital granted approval for all the animal experiments involved in this study, as documented by approval number 2023 − 564. All surgical interventions were meticulously executed in alignment with the established animal care guidelines. Male ICR mice, aged between 4 and 5 weeks and weighing approximately 18–20 g, were selected to establish a model for wound infection research. The procedure initiated with the careful removal of fur from the mice’s backs. This was followed by the creation of an 8 mm diameter wound on each mouse, which was subsequently inoculated with 10 µL of a bacterial solution at a concentration of 2 × 10^8^ CFU/mL. On the second day post-wounding, the mice were systematically categorized into four experimental groups: control (Ctrl), PADM, PADM@Mg, and PADM@MgC, with each group comprising eight mice. In brief, MRSA-infected wounds were administered a single treatment of either control (PBS), PADM, PADM@Mg, or PADM@MgC during the entire experimental period. The mice were provided unrestricted access to food and water. They were housed in microinsulators under regulated conditions of humidity, temperature, and a 12-hour light-dark cycle. Daily digital photographs of the wounds were captured, and the wound area was quantitatively assessed using Image J software. The percentage of wound closure was calculated using the following formula:

3 $$\eqalign{& {\rm{Scratch}}\,{\rm{open}}\,{\rm{area }}\left( \% \right){\rm{ }} \cr & = {\rm{ }}\left( {{\rm{remaining}}\,{\rm{wound}}\,{\rm{area/original}}\,{\rm{wound}}\,{\rm{area}}\,{\rm{vehicle}}} \right){\rm{ }} \times {\rm{ }}100 \cr}$$

### Microbiological and histological examination

Upon euthanization of the mice on day 4 post-surgery, the infected tissues were meticulously harvested and homogenized in 5 mL of PBS for bacterial load assessment, with bacterial quantification executed as previously described. Subsequently, both wound and adjacent tissues were procured for comprehensive histological analysis. This included the application of Hematoxylin and Eosin (HE) staining and Giemsa staining to evaluate tissue morphology and infection status. Additionally, immunofluorescence techniques were employed to ascertain the expression levels of endogenous markers iNOS and CD206, providing insights into the inflammatory and vascular responses within the tissue.

### Statistical analysis

Quantitative data were presented as the mean ± standard deviation (SD), except where noted differently. For the comparison between two distinct groups, a two-tailed Student’s t-test was employed. In cases involving multiple groups, analyses were performed using one-way analysis of variance (ANOVA), complemented by Tukey’s post hoc test for in-depth comparisons. All data calculations and statistical analyses were meticulously conducted using Excel 2016 and GraphPad Prism 9. The threshold for statistical significance was established at *P* < 0.05.

### Electronic supplementary material

Below is the link to the electronic supplementary material.

Supplementary Material 1

## Data Availability

No datasets were generated or analysed during the current study.
