# Supplementary material for: Multifunctional biomimetic hydrogel dressing provides anti-infection treatment and improves immunotherapy by reprogramming the infection-related wound microenvironment
Source: J Nanobiotechnology. 2024 Feb 28;22:80. doi: 10.1186/s12951-024-02337-3 (PMC10902999; doi:10.1186/s12951-024-02337-3)
Supplement: Supplementary file 1 — Supplementary Material 1 [file 12951_2024_2337_MOESM1_ESM.docx]

**Supporting Information**

**Multifunctional Biomimetic Hydrogel Dressing Provides Anti-Infection Treatment and Improves Immunotherapy by Reprogramming the Infection-Related Wound Microenvironment**

*Xiaogang Bao^#, 1^, Shicheng Huo^#, *, 1^, Zhenhua Wang^#, 2^, Shengyan Yang^3^, Linyun Dou^1^, Yifei Liu^1^, Jian Huang^1^, Chang Cai^1^, Bin Fang^*, 4^, Guohua Xu^*, 1^*

^1^ Department of Orthopedic Surgery, Spine Center, Changzheng Hospital, Navy Medical University, Shanghai 200003, China

^2^ Department of Laboratory Medicine, Shanghai Changzheng Hospital, Naval Medical University, Shanghai, 200003, China

^3^ Department of Pharmacy, Second Affiliated Hospital of Naval Medical University, Shanghai, China

^4^ Department of Orthopedics, the First Affiliated Hospital of Zhejiang Chinese Medical University, Hangzhou, 310000, China.

*Corresponding authors:

E-mail: xuguohuamail@smmu.edu.cn (Guohua Xu), waznxyz@126.com (Shicheng Huo), fangbin008@outlook.com (Bin Fang)

Xiaogang Bao#, Shicheng Huo#, and Zhenhua Wang #contributed equally to this work.

**1. Methods**

**1.1. Synthesis of Mg-MBG**

To prepare Mg-containing mesoporous bioglass nanospheres (Mg-MBG), a modified microemulsion-assisted sol-gel method was employed. To begin, 0.6 g of cetrimonium bromide (CTAB) was dissolved in 30 mL of deionized water at 60°C with rapid stirring. Once the solution cleared, 8 mL of ethyl acetate was added. After stirring for 20 minutes, 6 mL of ammonia solution (NH3⋅H2O, 1 M, VWR, Radnor, PA, USA) was added. Following another 20 minutes of stirring, 2.88 mL of tetraethyl orthosilicate (TEOS) and calcium nitrate tetrahydrate were sequentially added at 30-minute intervals. Magnesium nitrate was then added to the suspension and allowed to react for 4 hours. The resulting colloids were collected by centrifugation at 8000 ×g for 10 minutes and washed twice with deionized water and 99% ethanol. The collected deposits were dried at 60°C overnight before being calcined at 750°C for 3 hours to obtain Mg-MBG.

**1.2. Drug loading and release**

In brief, 5 mg of Cur was mixed with 50 mg of Mg-MBG in 50 mL of Tris-HCl (pH 8.8) and stirred in the dark at room temperature for 12 hours. Excess reagents were removed after centrifugation at 8000 ×g for 10 minutes, resulting in Mg-MBG@Cur. Drug release studies were conducted in phosphate buffer solution (pH 7.4). The phosphate buffer solution was prepared by mixing 13.9 g of potassium dihydrogen phosphate and 2.7 g of dipotassium phosphate in 1 liter of deionized water. The pH of the buffer medium was measured using pH paper. Samples were immersed in 4 mL of buffer solution contained in a glass bottle, then placed in a shaker at 150 rpm and 37°C to simulate human body movement. Each treatment was replicated three times. From day 1-10, buffer medium was collected from the glass bottles to measure drug release and replaced with 4 mL of fresh buffer medium. The optical density of the collected buffer medium was obtained using a UV-visible spectrophotometric microplate reader to analyze drug release. The absorbance value of Cur was 427 nanometers. Subsequently, drug concentration was measured using a standard curve, and a graph of cumulative release percentage versus time was plotted.

**Figure S1.** Comparison of DNA content in porcine skin before and after decellularization.


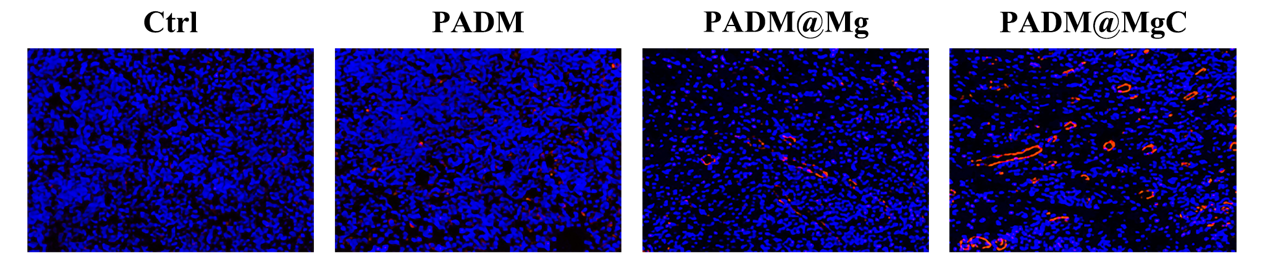


**Figure S2.** Representative immunofluorescence staining images of CD31 of wound skin tissues on day 10.

**Table S1** Primers used in this assay

| Gene and primer direction | Primer sequence (5′to 3′) |
| --- | --- |
| Mouse IL-1β |  |
| Forward | GCAACTGTTCCTGAACTCAACT |
| Reverse | ATCTTTTGGGGTCCGTCAACT |
| Mouse iNOS |  |
| Forward | GTTCTCAGCCCAACAATACAAGA |
| Reverse | GTGGACGGGTCGATGTCAC |
| Mouse IL-6 |  |
| Forward | TAGTCCTTCCTACCCCAATTTCC |
| Reverse | TTGGTCCTTAGCCACTCCTTC |
| Mouse CD206 |  |
| Forward | TACTTGGACGGATAGATGGAGG |
| Reverse | CATAGAAAGGAATCCACGCAGT |
| Mouse Actin |  |
| Forward | GGCTGTATTCCCCTCCATCG |
| Reverse | CCAGTTGGTAACAATGCCATGT |
